# Supplementary material for: Ten-year natural history of visual function in Japanese patients with Leber hereditary optic neuropathy: A retrospective cohort study
Source: PLoS One. 2026 Apr 28;21(4):e0348093. doi: 10.1371/journal.pone.0348093 (PMC13123968; doi:10.1371/journal.pone.0348093)
Supplement: S1 Table — Counts are shown as n (%). Supportive medication indicates receipt of at least one of the following: vitamins/supplements (including coenzyme Q10 preparations) and/or vasodilators. Regimens were heterogeneous and not protocolized. No patients received idebenone or gene therapy. (DOCX) [file pone.0348093.s004.docx]

**S1 Table. Exposure to non-specific supportive medications during follow-up.**

| **Medication exposure** | **Overall (N=27), n (%)** |
| --- | --- |
| Any supportive medication (≥1) | 21 (77.8%) |
| Vitamins/supplements | 18 (66.7%) |
| Coenzyme Q10 preparations | 16 (59.3%) |
| Vasodilators | 5 (18.5%) |
