# Supplementary material for: Ten-year natural history of visual function in Japanese patients with Leber hereditary optic neuropathy: A retrospective cohort study
Source: PLoS One. 2026 Apr 28;21(4):e0348093. doi: 10.1371/journal.pone.0348093 (PMC13123968; doi:10.1371/journal.pone.0348093)
Supplement: S2 Table — Data are presented as the mean (standard deviation), median (interquartile range). The subgroup consists of patients with the m.11778G > A mutation who were ≥15 years of age at onset. Abbreviations: BCVA, best-corrected visual acuity; M, months. (DOCX) [file pone.0348093.s005.docx]

| **S2 Table. Summary of BCVA** | | | |
| --- | --- | --- | --- |
|  |  | **All** | **Subgroup** |
| **12M BCVA,logMAR** | **Average** | **1.63 (0.51)** | **1.71 (0.41)** |
|  | **Median** | **1.70 (1.37,2.00)** | **1.70 (1.52, 2.00)** |
| **24M BCVA,logMAR** | **Average** | **1.56 (0.63)** | **1.67 (0.51)** |
|  | **Median** | **1.70 (1.22, 2.00)** | **1.70 (1.52, 2.00)** |
| **36M BCVA,logMAR** | **Average** | **1.54 (0.66)** | **1.65 (0.52)** |
|  | **Median** | **1.70 (1.15, 2.00)** | **2.00 (1.52, 2.00)** |
| **48M BCVA,logMAR** | **Average** | **1.50 (0.66)** | **1.62 (0.53)** |
|  | **Median** | **1.70 (1.05, 2.00)** | **1.70 (1.40, 2.00)** |
| **60M BCVA,logMAR** | **Average** | **1.51 (0.65)** | **1.64 (0.52)** |
|  | **Median** | **1.70 (1.10, 2.00)** | **2.00 (1.40, 2.00)** |
| **120M BCVA,logMAR** | **Average** | **1.53 (0.65)** | **1.66 (0.51)** |
|  | **Median** | **1.70 (1.22, 2.00)** | **2.00 (1.40, 2.00)** |
