# Supplementary material for: Ten-year natural history of visual function in Japanese patients with Leber hereditary optic neuropathy: A retrospective cohort study
Source: PLoS One. 2026 Apr 28;21(4):e0348093. doi: 10.1371/journal.pone.0348093 (PMC13123968; doi:10.1371/journal.pone.0348093)
Supplement: S3 Table — Piecewise linear mixed-effects models were refit after restricting the cohort to m.11778G > A cases. Monthly slope estimates were converted to annual rates by multiplying by 12. (DOCX) [file pone.0348093.s006.docx]

**S3 Table. Sensitivity analysis of BCVA slopes restricted to m.11778G>A cases**

| **Phase** | **Slope (logMAR/year)** | **95% CI** | **t value** |
| --- | --- | --- | --- |
| Chronic (12–60 months) | -0.027 | -0.043 to -0.012 | -3.379 |
| Late chronic (60–120 months) | 0.003 | -0.008 to 0.015 | 0.6 |
