# Supplementary material for: Ten-year natural history of visual function in Japanese patients with Leber hereditary optic neuropathy: A retrospective cohort study
Source: PLoS One. 2026 Apr 28;21(4):e0348093. doi: 10.1371/journal.pone.0348093 (PMC13123968; doi:10.1371/journal.pone.0348093)
Supplement: S4 Table — BCVA (logMAR), GP grade, and CFF are shown at 12, 60, and 120 months from onset. Unit of analysis is the eye; some patients contributed both eyes. Because GP and CFF were not protocolized and were typically assessed at ~24-month intervals in routine practice, this table is descriptive and hypothesis-generating, and was not used to support the primary conclusions. (DOCX) [file pone.0348093.s007.docx]

**S4 Table. Descriptive cross-modality trajectories in eyes with complete BCVA, Goldmann perimetry grade, and critical flicker frequency data (6 patients; 11 eyes).**

|  | **BCVA, logMAR** | | | **GP grade** | | | **CFF, Hz** | | |
| --- | --- | --- | --- | --- | --- | --- | --- | --- | --- |
|  | **12M** | **60M** | **120M** | **12M** | **60M** | **120M** | **12M** | **60M** | **120M** |
| Eye 1 | 2.000 | 1.699 | 1.699 | 1.5 | 2 | 2.5 | 10 | 17 | 17 |
| Eye 2 | 2.000 | 2.000 | 2.000 | 1.5 | 3 | 2.5 | 10 | 21 | 30 |
| Eye 3 | 2.000 | 2.000 | 2.000 | 2 | 3 | 3 | 13 | 15 | 34 |
| Eye 4 | 1.699 | 1.699 | 1.699 | 2 | 2.5 | 2.5 | 10 | 17 | 15 |
| Eye 5 | 1.699 | 1.523 | 1.699 | 3 | 2.5 | 2 | 23 | 20 | 21 |
| Eye 6 | 2.301 | 2.000 | 2.000 | 2.5 | 2.5 | 2.5 | 13 | 8 | 11 |
| Eye 7 | 1.699 | 2.000 | 2.000 | 2 | 2.5 | 2.5 | 12 | 16 | 14 |
| Eye 8 | 1.398 | 1.000 | 1.000 | 1 | 1.5 | 1.5 | 13 | 21 | 21 |
| Eye 9 | 1.699 | 1.097 | 1.000 | 1 | 1.5 | 1.5 | 12 | 28 | 20 |
| Eye 10 | 1.000 | 2.000 | 2.000 | 2 | 4 | 4 | 11 | 12 | 13 |
| Eye 11 | 2.000 | 2.000 | 2.000 | 2.5 | 4 | 4 | 8 | 11 | 11 |
