# Supplementary figures and images for: Ten-year natural history of visual function in Japanese patients with Leber hereditary optic neuropathy: A retrospective cohort study
Source: PLoS One. 2026 Apr 28;21(4):e0348093. doi: 10.1371/journal.pone.0348093 (PMC13123968; doi:10.1371/journal.pone.0348093)

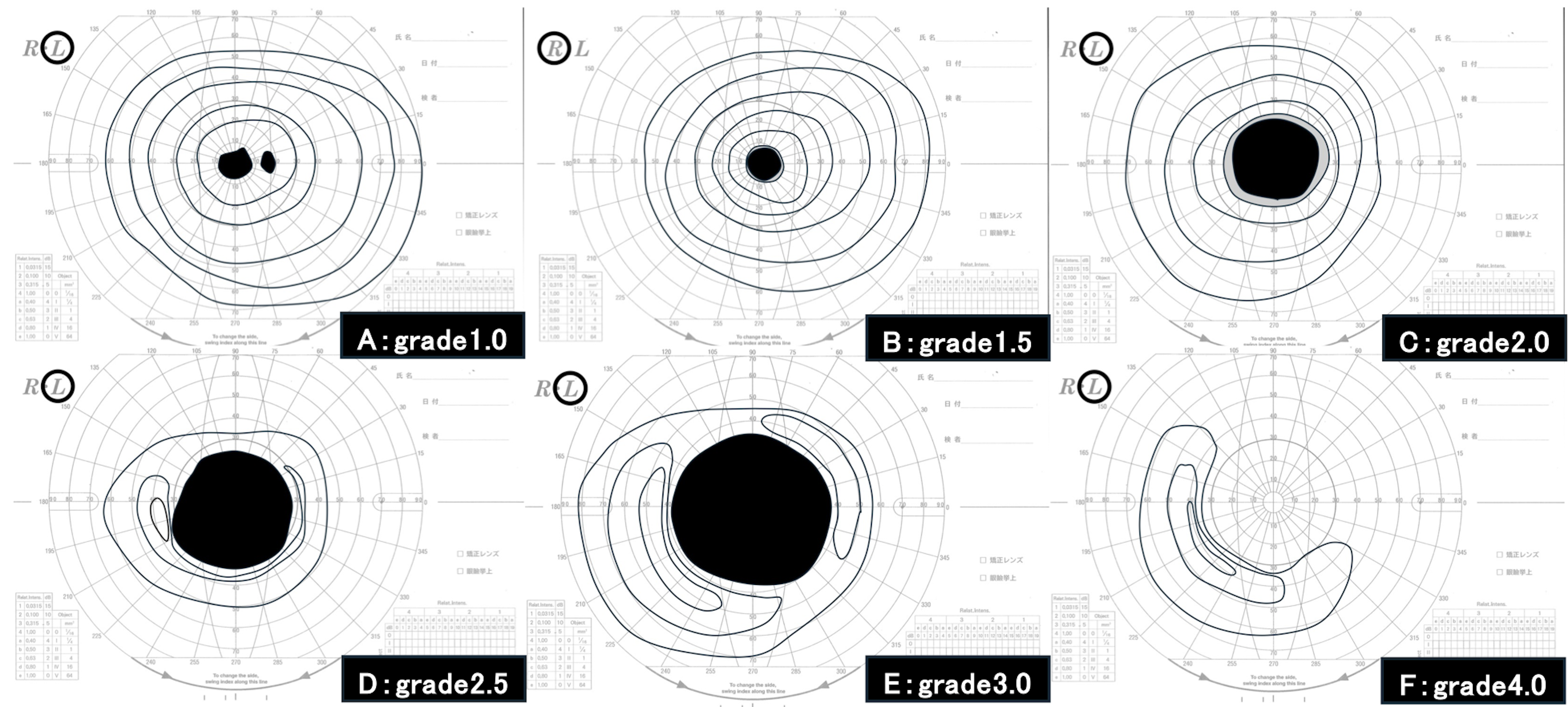

Supplement: S1 Fig — Visual field defect grades were defined as follows: Grade 0, normal field; Grade 1, central scotoma within 10°; Grade 2, central scotoma 10–30°; Grade 3, central scotoma ≥30°; and Grade 4, only the peripheral field remaining. The grade was further refined by adding 1.0 point if the scotoma extended more than halfway beyond a prespecified boundary and 0.5 point if it extended halfway or less. Examples are shown for A, Grade 1.0; B, Grade 1.5; C, Grade 2.0; D, Grade 2.5; E, Grade 3.0; and F, Grade 4.0. (TIF) [file pone.0348093.s008.tif]

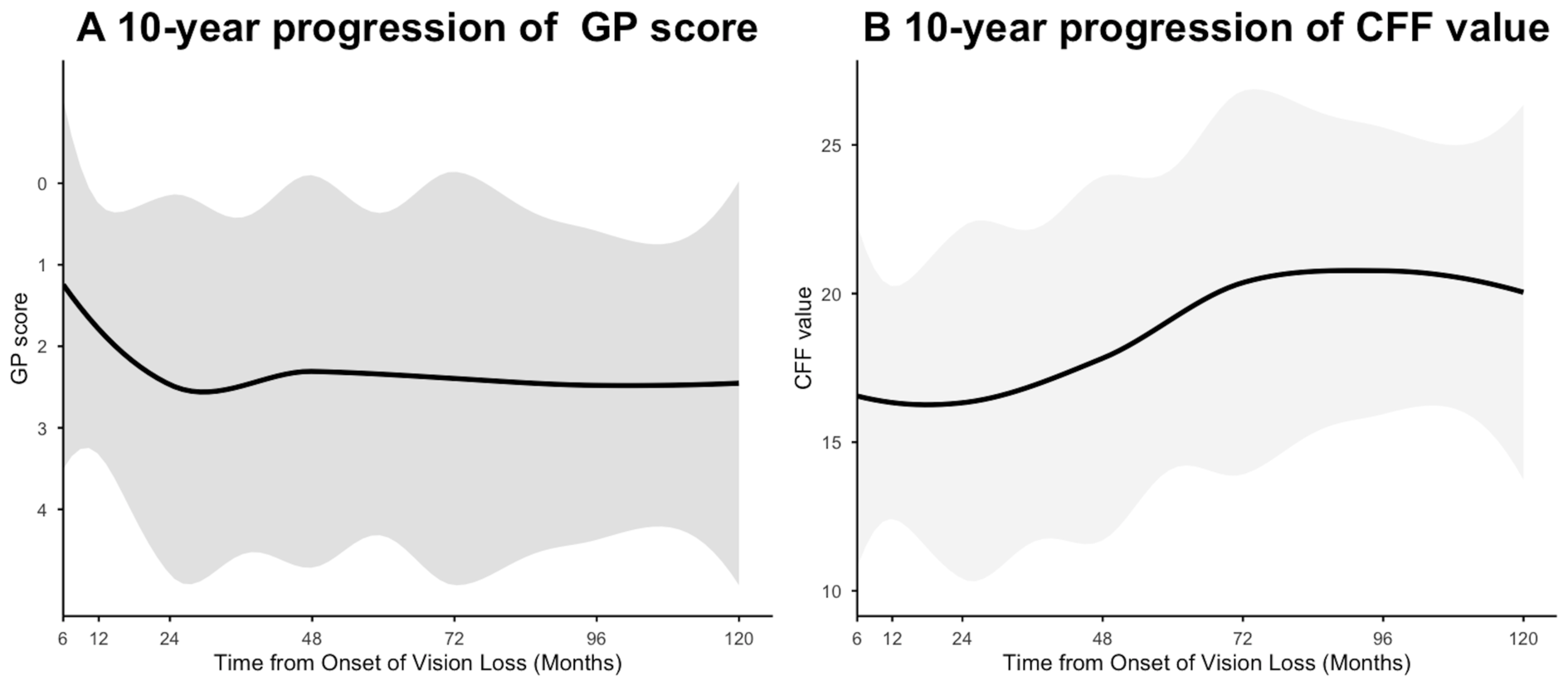

Supplement: S2 Fig — Common to all panels. The x-axis shows the months after onset. The y-axis shows the GP score for (A; higher values indicate more severe impairment) and CFF (Hz) for (B). Points and solid lines depict the mean values at each time point, and shaded bands indicate 95% confidence intervals (CIs). LOWESS (local regression) was used for descriptive visualization. See Methods for smoothing parameters (e.g., span). (A) Visual field: LOWESS-smoothed curve with a 95% confidence band (10 patients; 19 eyes; 132 assessments). (B) CFF: LOWESS-smoothed curve with a 95% confidence band (11 patients; 19 eyes; 137 assessments). Note. No inferential modeling was performed for these outcomes. (TIF) [file pone.0348093.s009.tif]
