# Supplementary material for: Ten-year natural history of visual function in Japanese patients with Leber hereditary optic neuropathy: A retrospective cohort study
Source: PLoS One. 2026 Apr 28;21(4):e0348093. doi: 10.1371/journal.pone.0348093 (PMC13123968; doi:10.1371/journal.pone.0348093)
Supplement: Attachment — Submitted filename: Review.pdf [file pone.0348093.s011.pdf]

General Comments

This study provides rare, long-term (≥10 years) follow-up of Japanese LHON patients, describing phase-specific BCVA trajectories. Strengths include genetically confirmed diagnoses and robust mixed-effects modeling. Limitations include small sample size, selection bias, and limited exploratory analyses. Overall, the manuscript offers valuable descriptive benchmarks for future studies.

Introduction

- Clarity of gap: While the rationale for long-term follow-up is stated, the manuscript could more explicitly emphasize why Asian cohorts might differ from Western cohorts (genetic, environmental, or healthcare-related factors).
- Overlap with previous studies: The introduction references Lam et al. and other natural-history studies but could more clearly differentiate how this study's design (near-onset enrollment, 10-year follow-up) uniquely contributes new knowledge.
- Objective phrasing: Objectives are clear, but "assess consistency in a predefined ≥15 years, m.11778G>A subgroup" could be rephrased for clarity; it's slightly ambiguous whether this refers to age, mutation, or both.

Methods

- Selection bias: Only patients with ≥10 years follow-up were included, potentially overrepresenting patients with milder or more stable disease. This could bias results toward apparent late-phase stability.
- Sample size and subgroup limitations: The final analytic cohort (27 patients, 53 eyes) is small, particularly for subgroup and exploratory analyses (GP, CFF), limiting statistical power.
- BCVA conversion: Defining counting fingers, hand motion, and light perception as specific logMAR values is standard, but the rationale could be cited explicitly to justify comparisons over 10 years.
- Kaplan–Meier analyses: Not accounting for delayed entry or clustering by patient may over- or underestimate cumulative probabilities. This should be clearly acknowledged as a limitation in Methods.
- Missing data handling: Linear mixed-effects models assume missing-at-random; it would help to discuss how deviations from this assumption could influence slope estimates, especially given variable follow-up intensity.

Results

- Presentation of slopes: Chronic-phase improvement is statistically significant but small (~0.03 logMAR/year); the clinical relevance should be emphasized.
- Late Chronic phase: Confidence intervals are wide, and the absence of a trend may reflect low power rather than true stability.
- Kaplan–Meier curves: Descriptive, but survival function never falls below 0.5; the lack of median times should be explicitly tied to small sample size and follow-up limitations.
- Exploratory measures (GP, CFF): Data are highly limited; interpretations should remain clearly hypothesis-generating. Presenting mean ± SD without longitudinal modeling may overstate apparent trends.
- Repetition: Some statements about plateauing after 60 months and cumulative probability curves are repeated; streamlining would improve clarity.

Discussion

- Interpretation of ethnic differences: The suggestion that Japanese/Asian patients may have better outcomes is interesting but speculative. Confounding factors (environment, maternal haplogroups, healthcare access) are mentioned but could be emphasized more strongly to avoid overinterpretation.
- Comparison with Western cohorts: Direct comparisons are limited by design differences; this could be highlighted earlier in the discussion.
- Clinical significance: The magnitude of chronic-phase improvement is small; discuss what this means for patient counseling or prognosis.
- Limitations: Well described, but the impact of selection bias on apparent late-phase stability could be reinforced.
- Generalisability: Findings from a single tertiary centre with selected long-term attendees may not apply to broader incident LHON populations. This is noted but could be more strongly linked to the study's descriptive nature.

Conclusion

BCVA showed modest improvement during the Chronic phase (12–60 months) and no clear trend in the Late Chronic phase (60–120 months). These findings serve as

descriptive, phase-specific benchmarks rather than definitive evidence of long-term stability or ethnic differences.
