# Supplementary material for: Ten-year natural history of visual function in Japanese patients with Leber hereditary optic neuropathy: A retrospective cohort study
Source: PLoS One. 2026 Apr 28;21(4):e0348093. doi: 10.1371/journal.pone.0348093 (PMC13123968; doi:10.1371/journal.pone.0348093)
Supplement: Attachment — Submitted filename: Review.pdf [file pone.0348093.s013.pdf]

General

- Small sample size and selection bias limit generalizability.
- Wide confidence intervals reduce certainty of long-term trends.
- Some sentences across sections are overly long, affecting readability.

Introduction

- Could be more concise, some sentences are long and dense.
- Lacks a clear, explicit statement of study hypothesis or primary objective.

Methods

- Rationale for phase divisions (Acute, Chronic, Late Chronic) should be briefly explained.
- Inclusion/exclusion criteria and handling of missing data are not fully detailed.
- Dense sentences (e.g., 117–128) reduce readability; consider splitting.
- Statistical software/package not specified.

Results

- What is Nadir BCVA? 48M and 120M?
- Dense numeric reporting; tables or figures could improve clarity.
- Percentages of eyes vs. patients sometimes unclear.
- Figures (LOWESS, Kaplan–Meier) need clearer referencing and brief interpretation in text.
- Functional significance of BCVA changes could be emphasized.
- Some numerical results could be presented in a table for clarity rather than embedded in dense text.
- Make it clear when percentages refer to eyes vs. patients to avoid confusion.
- Consider emphasizing functional significance (e.g., BCVA ≤1.6 logMAR = threshold for legal blindness in Japan).

Discussion

- There could be improvement in visual acuity after certain time period across all LHON types, which was not addressed adequately

- Some interpretations may overstate findings; use more cautious phrasing.
- Limited discussion on potential biological/environmental reasons for differences from Western cohorts.
- Clinical relevance of small chronic-phase improvements could be elaborated.
