# Supplementary material for: Ten-year natural history of visual function in Japanese patients with Leber hereditary optic neuropathy: A retrospective cohort study
Source: PLoS One. 2026 Apr 28;21(4):e0348093. doi: 10.1371/journal.pone.0348093 (PMC13123968; doi:10.1371/journal.pone.0348093)
Supplement: Attachment — Submitted filename: Respond_to_Reviewer.docx [file pone.0348093.s012.docx]

Dear Editor,

Thank you for the opportunity to submit a revised version of our manuscript. We have carefully addressed the journal requirements and the reviewers’ comments. Below we summarize how we have complied with the additional journal requirements:

1. PLOS ONE style requirements and file naming

We revised the manuscript to comply with PLOS ONE style requirements, including formatting and file naming. We moved figure captions and table into the main text at first citation (read order) and revised the in-text citation format from superscript numbers to bracketed numbers [ ], consistent with PLOS ONE requirements. We are submitting (i) a clean revised manuscript and (ii) a marked-up version with changes highlighted, along with appropriately named figure and Supporting Information files.

2. Author information (Yasuyuki Takai)

We have amended the manuscript submission data (via “Edit Submission”) to include Yasuyuki Takai as an author, as requested.

3. Supporting Information captions and in-text citations

We included captions for all Supporting Information files at the end of the manuscript and updated the in-text citations to ensure consistency with the Supporting Information labels (S1 Table, S2 Fig, etc.), following the journal’s Supporting Information guidelines.

4. Replacement of S1 Fig

We uploaded a new version of S1 Fig with improved clarity and resolution to meet the figure requirements.

In addition, we updated Figure 1 (study flow) to explicitly report the number of excluded cases by each eligibility criterion, improving transparency of the selection process. We also moved the former Supporting Information Table 1 into the main manuscript as Table 2 to better highlight key results in the Results section and to keep the Supporting Information concise as the number of supplementary items increased.

We also reviewed the reference list to ensure completeness and correctness. We did not intentionally cite any retracted articles.

We appreciate your and the reviewers’ time and consideration, and we hope that the revised manuscript is now suitable for further evaluation.

Sincerely,

Yasuyuki Takai, MD/PhD

on behalf of all authors

**Reviewer #1 – Response (green characters)**

Comment 1: Selection/attrition bias; cross-sectional data; suggested paper reporting improvement

Response:

Thank you for this important comment. We agree that attrition due to visual improvement is a potential source of selection bias in long-term retrospective cohorts. We have clarified in the Limitations that we did not have protocolized cross-sectional outcome data for patients who did not meet the ≥10-year follow-up criterion and that our results should therefore be interpreted as descriptive benchmarks among long-term attendees. In addition, we added a sentence (with a supporting citation) noting that clinically meaningful visual improvement has been reported in a subset of LHON patients and that early improvers may be underrepresented in long-term follow-up cohorts (Kim et al., 2024).

Manuscript changes:

- Discussion (p20, line 345-352): “We did not have protocolized cross-sectional outcome data for patients who did not meet the 10-year follow-up criterion; thus, selection related to long-term follow-up cannot be excluded and the present trajectories should be interpreted as descriptive benchmarks for long-term attendees. Visual improvement has been reported in a subset of LHON patients and early improvers may be underrepresented in long-term follow-up cohorts [14]. Patients with early visual improvement may be underrepresented due to the ≥10-year follow-up requirement; thus, our estimates should be interpreted as benchmarks among long-term attendees rather than population-level improvement rates.”

Comment 2: Why 53 eyes instead of 54 eyes in 27 patients?

Response:

Among 27 patients (54 eyes), one eye developed retinal vein occlusion during follow-up, which could confound BCVA trajectories. As prespecified in the revision, this eye was excluded from BCVA analyses, resulting in 53 analyzed eyes.

Manuscript changes:

- Materials and methods (p5, lines 83-86) “After applying these patient-level criteria, 27 patients were included. At the eye level, among 54 eyes from these 27 patients, one eye developed retinal vein occlusion during follow-up and was excluded from BCVA analyses, resulting in 53 eyes for the primary analyses (Fig 1).”

Comment 3: Idebenone supplementation and other medications potentially influencing outcomes

Response:

No patients received idebenone or gene therapy during follow-up. Some patients received non-specific/off-label supportive medications (e.g., vitamins/supplements including coenzyme Q10 preparations and/or vasodilators); regimens were heterogeneous and not protocolized. We therefore did not attempt causal inference regarding these medications and clarified this point in the manuscript. Supporting medication exposure is summarized in the Supporting Information.

Manuscript changes:

- Materials and methods (p5, lines 86-89) “No patients received idebenone or gene therapy during follow-up. Low-dose, non-specific off-label supportive medications (e.g., vitamins/supplements including coenzyme Q10 and occasional vasodilators) were used at clinicians’ discretion; regimens were heterogeneous and non-protocolized.”

- Added Supporting Information (S1 Table)

- Results (p11, line185-188) “Overall, 21/27 patients (77.8%) received at least one non-specific supportive medication during follow-up, vitamins/supplements (18/27, 66.7%) and coenzyme Q10 preparations (16/27, 59.3%); vasodilators were used in 5/27 patients (18.5%) (S1 Table).”

emphasized in the Limitations that these data are descriptive and not used to infer treatment effects.

- Discussion (p21, line 365-367):” Finally, because supportive medications were non-protocolized, we did not estimate treatment effects and present findings as descriptive benchmarks rather than treatment-response estimates.”

Comment 4: Mean age appears higher than in other studies—why?

Response:

We acknowledge that the mean age in our analytic cohort may appear higher than in some prior reports. This likely reflects the age distribution of LHON in contemporary Japanese cohorts reported in prior epidemiologic work, and our inclusion criterion requiring ≥10 years of continuous follow-up, which may preferentially capture patients with different healthcare utilization patterns (including older onset and/or long-term attendees at a tertiary center). We have emphasized this as part of the study’s generalizability limitations.

Manuscript changes:

- Discussion (p21, line 361-364): “Our cohort consisted exclusively of Japanese patients, and genetic or environmental background may differ from Western cohorts; age at onset was somewhat higher than in some earlier studies, consistent with contemporary Japanese epidemiology [15], although long-term follow-up eligibility may also have influenced our age distribution.”

Comment 5: Need more detailed “integrated” analysis across BCVA, visual field, and CFF (concordance/discordance)

Response:

We agree that cross-modality concordance and discordance would be informative. However, simultaneous/paired measurements of BCVA, Goldmann perimetry, and CFF at comparable time points were available in only a small subset. Because of this limited overlap, we did not model integrated outcomes formally. Instead, we added a hypothesis-generating descriptive summary limited to eyes with complete data across modalities, clearly labeled as exploratory.

Manuscript changes:

- Result (p21, line 297-299): “Within-eye longitudinal changes across modalities were not always concordant; S4 Table is provided descriptively as hypothesis-generating and was not used to support the primary conclusions.”

- Added a Supporting Information table (S4 table)

Comment 6: Too few cycles for visual field and CFF to draw conclusions

Response:

We agree. This limitation was already stated in the original manuscript (Methods section), and we have further clarified it in the revised version by explicitly noting the limited power/variability in the ancillary GP and CFF subsets (Limitations). These results remain descriptive and hypothesis-generating and were not used to support the primary BCVA trajectory conclusions.

Manuscript changes:

- Discussion (p20, line 357 – p21, line 361): “The sample size was modest, particularly in the late chronic phase and the ancillary GP and CFF subsets, resulting in wide confidence intervals and limited power; therefore, the absence of a statistically significant late-phase slope should not be interpreted as definitive stability, and visit-to-visit fluctuations also related to exertion, or fatigue may further increase measurement variability.”

**Reviewer #2 – Response (orange characters)**

Comment 1: Unclear phrasing regarding delayed entry in Kaplan–Meier curves (lines 115–116)

Response:

Thank you for pointing this out. We agree that the original phrasing was unclear and could be interpreted as implying that the absence of delayed entry was caused by post-onset enrolment. We have rephrased this sentence to state more precisely that the Kaplan–Meier analyses were conducted without implementing delayed entry (left truncation), and that the resulting curves should be interpreted as descriptive summaries with this limitation in mind.

Manuscript changes:

- Materials and methods (p8, lines 136-138) “Because we did not implement delayed entry (left truncation) in these Kaplan–Meier analyses, early cumulative attainment may be biased if onset-to-enrolment times differ systematically.”

Comment 2: Please include a paragraph about the clinical significance of CFF and its intended information

Response:

We appreciate this suggestion. We have added a dedicated paragraph describing the clinical rationale for CFF in optic neuropathies. Specifically, we clarify that CFF is a psychophysical measure of temporal resolution that reflects afferent visual pathway function, has been used as a functional marker in optic nerve disorders, and has been incorporated as an ancillary outcome in prior LHON literature. We also emphasize that CFF was analyzed as an exploratory/ancillary measure in our study given limited, non-protocolized longitudinal measurements.

Manuscript changes:

- Materials and methods (p9, lines 160-164) “CFF is a psychophysical index of visual temporal resolution and has been used as a practical functional marker of afferent visual pathway integrity in optic nerve disease [9]. Prior LHON studies have incorporated CFF improvement into recovery criteria, supporting its role as a complementary endpoint to BCVA and perimetry [10].”

Comment 3: “Based on only 10–11 patients” is odd; you already state 11 patients tested

Response:

Thank you for catching this inconsistency. We have revised the text to report the sample sizes explicitly and consistently: Goldmann perimetry data were available for 10 patients, whereas CFF data were available for 11 patients. We removed the approximate “10–11” phrasing to avoid ambiguity.

Manuscript changes:

- Discussion (p19, lines 338-339) “However, these analyses were based on only 10 patients for GP and 11 patients for CFF with unbalanced follow-up and substantial variability, and the models…”

**Reviewer #3 – Response (purple characters)**

General comment

Response:

We sincerely thank the reviewer for the constructive and detailed critique. We agree with the key points regarding the descriptive value of this rare ≥10-year Japanese cohort, as well as the main limitations (small sample size, selection bias, and limited exploratory data for GP/CFF). In the revision, we refined the framing throughout to emphasize that our findings provide phase-specific descriptive benchmarks among long-term attendees, rather than definitive evidence of long-term stability or ethnic differences.

Comment: Clarify why Asian cohorts might differ from Western cohorts

Response:

We agree. We revised the text to more explicitly state plausible reasons why long-term outcomes may differ across cohorts (e.g., genetic background including mitochondrial haplogroups, environmental factors, and healthcare access/utilization), while maintaining a cautious, non-causal tone.

Manuscript changes:

- Introduction (p4, lines 64-66) “Data on long-term visual trajectories in Asian LHON cohorts remain limited, and cross-cohort differences may reflect variation in mitochondrial genetic background (e.g., haplogroup distributions), environmental exposures, and healthcare access.”

Comment: Differentiate this study from previous natural-history studies

Response:

We agree that the unique contribution should be clearer. We revised the Introduction to better distinguish our design—near-onset enrollment with continuous ≥10-year follow-up and phase-specific trajectory modeling using a prespecified piecewise mixed-effects framework—from prior studies with different enrollment windows and/or shorter follow-up.

Manuscript changes:

- Introduction (p4, lines 71- p5 line 75) “In contrast to prior natural-history reports that often include heterogeneous enrolment timing and variable follow-up completeness, our study focuses on a near-onset cohort (presentation within 6 months) with continuous follow-up for ≥10 years at a single tertiary center, enabling phase-specific longitudinal estimates over the chronic and late chronic periods.”

Comment: Objective phrasing is ambiguous (“predefined ≥15 years, m.11778G>A subgroup”)

Response:

Thank you. We clarified that this prespecified subgroup is defined by both onset age ≥15 years and the primary mutation m.11778G>A, to avoid ambiguity.

Manuscript changes:

- Introduction (p4, lines 70-71) “….. and (iii) assess consistency in a predefined subgroup defined as onset age ≥15 years and m.11778G>A subgroup [5].”

Comment: Selection bias (≥10-year follow-up may overrepresent milder/stable disease; may bias toward apparent late-phase stability)

Response:

We agree and have reinforced this limitation. We now emphasize that restricting to ≥10-year continuous follow-up may underrepresent patients who discontinue follow-up (including those with early improvement) and may also enrich for long-term attendees with different disease/healthcare utilization patterns. We therefore frame late-phase “no clear trend” as a descriptive finding in this selected cohort, not proof of true long-term stability.

Manuscript changes:

- Strengthened limitation statements regarding selection/attrition bias and its potential impact on late-phase trajectories.

Comment: Small sample size and subgroup/exploratory limitations (GP/CFF)

Response:

We agree. This is stated in the Materials and methods section in the GP/CFF methods description, where GP/CFF are explicitly characterized as “ancillary and exploratory” and positioned as hypothesis-generating rather than supporting the primary conclusions. The same positioning is carried into the Results, where the GP/CFF analyses are presented descriptively with explicit caution regarding the limited sample size and irregular timing. It is reiterated in the Discussion, which notes the small ancillary subcohorts, limited power, and the descriptive/hypothesis-generating nature of GP/CFF findings.

Manuscript changes:

- No change

Comment: BCVA conversion (CF/HM/LP) should be explicitly justified/cited

Response:

We agree and added an explicit citation supporting the standard logMAR conversions for CF/HM/LP used in longitudinal comparisons. We added the following reference (Reference.7 Lange et al) to support the conversion of the clinical acuity categories “hand motion” and “counting fingers” to logMAR values:

Manuscript changes:

- Materials and methods (p7, lines 112-114). “For very low vision, counting fingers, hand motion, and light perception were converted to 2.0, 2.3, and 4.0 logMAR, respectively, consistent with prior LHON natural-history studies and established low-vision quantification methods [5-7].”

Comment: Kaplan–Meier analyses—lack of delayed entry and clustering should be clearly acknowledged in Methods

Response:

We agree. We clarified that Kaplan–Meier analyses were used as descriptive summaries and were conducted without implementing delayed entry (left truncation) or within-patient clustering, which may bias cumulative probabilities.

Manuscript changes:

- Materials and methods (p8, lines 136-138) “Because we did not implement delayed entry (left truncation) in these Kaplan–Meier analyses, early cumulative attainment may be biased if onset-to-enrolment times differ systematically.”

＊Following a similar point raised by another reviewer, the character is set to orange.

Comment: Missing data handling—LMM assumes missing at random; discuss deviations given variable follow-up intensity

Response:

We agree. We added text acknowledging that mixed-effects modeling assumes missing-at-random and that deviations (e.g., follow-up intensity related to unobserved outcomes) could influence slope estimates. This is particularly relevant in long-term retrospective follow-up.

Manuscript changes:

- Discussion (p20, lines 355-357) “Follow-up intensity varied and some data were missing; linear mixed-effects models used all available observations under a missing-at-random assumption, but bias remains possible if missingness depended on unobserved outcomes.”

Comment: Chronic-phase improvement is statistically significant but small; emphasize clinical relevance

Response:

Thank you for this helpful suggestion. We agree that the chronic-phase slope is statistically significant but modest in magnitude. This point is stated in the Results section in the paragraph beginning “In the linear mixed-effects model, during the Chronic phase (12–60 months)…,” where we describe a small but statistically significant improvement (−0.030 logMAR/year in the overall cohort and −0.025 logMAR/year in the subgroup). It is also emphasized in the opening paragraph of the Discussion beginning “Here, we characterized the long-term course of visual function…,” where we translate the slope into an approximate 5-year change and note that the magnitude is small and may have limited functional significance, with cautious counseling implications.

Manuscript changes:

- No change

Comment: Late chronic phase—wide CIs; “no trend” may reflect low power

Response:

We agree. As already noted in the manuscript, the late chronic-phase estimates have wide confidence intervals and limited power, and the absence of a detectable trend should not be interpreted as definitive stability. This is described in the Results section in the paragraph beginning “During the Late Chronic phase (60–120 months), the estimated slopes were close to zero…,” where we state that the confidence intervals are wide and remain compatible with slight long-term improvement or deterioration. The same interpretive caution is reiterated in the Discussion opening paragraph beginning “Here, we characterized…,” where we explain that the late-phase estimates are best interpreted as showing no clearly detectable directional trend rather than definitive stability. It is further reflected in the Limitations paragraph beginning “This study has several limitations…,” where we note limited power and that the absence of a statistically significant late-phase slope should not be interpreted as definitive stability.

Manuscript changes:

- No change

Comment: Kaplan–Meier curves never fall below 0.5; absence of median times should be explicitly tied to sample size/follow-up

Response:

We agree. This is stated in the Results section within the Kaplan–Meier description beginning “We estimated the cumulative probability of eyes achieving the prespecified BCVA thresholds…,” where we note that across all curves the survival function did not fall below 0.5 and therefore the median time to attainment could not be estimated, and we add that late-phase interpretation is limited by the small number of eyes at risk. The same rationale is also described in the Figure 3 legend in the sentence explaining that the survival function did not drop below 0.5 and thus the median time to attainment was not estimable.

Manuscript changes:

- No change

Comment: Exploratory GP/CFF—limited data; mean±SD without longitudinal modeling may overstate trends

Response:

We agree. This framing is stated in the Discussion section in the paragraph beginning “We also explored long-term trajectories of visual field defects and CFF…,” where we describe GP and CFF as ancillary/exploratory measures based on a small subset and caution against confirmatory interpretation. It is also reflected in the Results sections reporting GP/CFF (e.g., the CFF subsection beginning “For CFF, 11 patients…”), where the limited sample and descriptive nature of these analyses are made clear.

Manuscript changes:

- No change

Comment: Repetition—streamline repeated statements

Response:

We agree and have streamlined the Discussion to reduce repetition of the post–60-month plateauing description and the Kaplan–Meier interpretation. Specifically, we removed redundant re-statements of numerical results and consolidated the interpretation so that the Results section presents the quantitative findings, while the Discussion focuses on their interpretation and clinical implications.

Manuscript changes:

- Discussion (p17, lines 302-310) “Here, we characterized long-term visual trajectories in a Japanese LHON cohort with ≥10 years of follow-up at a single tertiary center. BCVA showed a modest improvement during the Chronic phase, whereas late-phase estimates did not show a clearly detectable directional trend and should not be interpreted as definitive stability given the wide confidence intervals and the declining number at risk over time. Taken together, these results provide phase-specific quantitative benchmarks for long-term attendees. Although chronic-phase improvement was statistically detectable, the magnitude was small and may have limited functional significance; clinically, recovery—when it occurs—appears typically modest and most likely within the first few years after onset.”

Comment: Ethnic differences are speculative; emphasize confounding (haplogroups/environment/healthcare)

Response:

We agree. This point was already addressed in the Discussion (ethnic differences paragraph beginning “Regarding ethnic differences…”), where we explicitly note that any apparent between-cohort differences are speculative and may reflect multiple confounders, including measurement differences, environmental exposures (e.g., smoking), and maternal haplogroup distribution. We continue to frame the present results primarily as single-center Japanese descriptive benchmarks.

Manuscript location:

- No change

Comment: Clinical significance for counseling/prognosis

Response:

We agree and have revised the Discussion to more explicitly describe the magnitude as modest in clinically interpretable terms (approximate change over five years) and to note the implications for counseling, while avoiding overgeneralization beyond this selected long-term cohort.

Manuscript changes:

- Discussion (p18, lines 307-310) “Although chronic-phase improvement was statistically detectable, the magnitude was small and may have limited functional significance; clinically, recovery—when it occurs—appears typically modest and most likely within the first few years after onset.”

**Reviewer #4 – Response** **(blue characters)**

Comment 1: Please provide information on the 3 patients with the m.14484 mutation separately, as spontaneous improvement may skew the results; consider reporting the other 24 patients separately.

Response:

Thank you for this important point. We agree that m.14484T>C can show substantial spontaneous recovery and that, in small cohorts, even a few such cases could influence aggregate estimates. Rather than presenting mutation-specific trajectories for the m.14484T>C subgroup (n=3), which would be underpowered and could be potentially misleading, we addressed this concern by conducting a sensitivity analysis restricted to the m.11778G>A subgroup, which represents the large majority of our cohort. The phase-specific BCVA slope patterns in this restricted analysis were consistent with the primary results, indicating that our main conclusions are not driven by the small m.14484T>C subgroup (S3 Table).

Manuscript changes:

- Materials and methods (p8, lines 129-130) “As a sensitivity analysis, we repeated the primary BCVA trajectory models after restricting the cohort to eyes with the m.11778G>A mutation.”

- Results (p13, lines 220-222) “To address the potential influence of the small m.14484T>C subgroup, we repeated the primary BCVA trajectory analyses restricted to m.11778G>A cases; the phase-specific patterns were similar (S3 Table).”

- Added a Supporting Information table (S3 table)

Comment 2: Figure 1 legend is too long and repeats the text; please shorten.

Response:

We agree and have shortened the Figure 1 legend to avoid repetition and to focus on the essential inclusion/exclusion flow.

Manuscript changes:

- Revised and condensed the Figure 1 legend.

**Reviewer #5 – Response (red characters)**

Comment 1: The statement that rarity hinders establishing appropriate control groups is “harsh”; idebenone and gene therapy studies have control groups. Please add relevant articles.

Response:

Thank you for this important point. We agree that controlled trials for LHON treatments have been conducted, including placebo-controlled idebenone trials and sham-/controlled gene therapy studies. We revised the relevant sentence to avoid implying that control groups cannot be established at all. Instead, we now state more precisely that the rarity and clinical heterogeneity of LHON can make it challenging to assemble adequately powered and broadly representative control groups across subpopulations and time windows in real-world settings, particularly for long-term comparisons. We also added citations to key controlled studies of idebenone and gene therapy, as suggested.

Manuscript changes:

- Introduction (p4, line 60-64) “Recently, disease-modifying therapies for LHON, including oral idebenone [3] and intravitreal gene therapy [4], have been investigated in controlled clinical trials. Although key studies have used randomized placebo- or sham-controlled or comparative designs, the rarity of LHON and heterogeneity in onset and follow-up make it difficult in practice to assemble control groups at adequate scale.”

Comment 2: Wide confidence intervals might reflect fatigue or pseudo-Uhthoff–like effects rather than small long-term changes.

Response:

We agree that within-subject fluctuation in visual function—including day-to-day variability, fatigue-related changes, and Uhthoff-like transient worsening reported in LHON—may contribute to variability in measured BCVA and could therefore widen confidence intervals, especially given irregular follow-up intervals and limited sample size. We have added this as an additional interpretive consideration in the Discussion. We also clarified that wide confidence intervals reflect both limited power and potential measurement/physiological variability; therefore, late chronic-phase estimates should be interpreted cautiously and should not be taken as definitive evidence of long-term stability or true absence of change.

Manuscript changes:

- Discussion (p20, line 357- p21, line 361) “The sample size was modest, particularly in the late chronic phase and the ancillary GP and CFF subsets, resulting in wide confidence intervals and limited power; therefore, the absence of a statistically significant late-phase slope should not be interpreted as definitive stability, and visit-to-visit fluctuations also related to exertion, or fatigue may further increase measurement variability.”

＊Following a similar point raised by another reviewer, the character is set to green.
