# Supplementary material for: Ten-year natural history of visual function in Japanese patients with Leber hereditary optic neuropathy: A retrospective cohort study
Source: PLoS One. 2026 Apr 28;21(4):e0348093. doi: 10.1371/journal.pone.0348093 (PMC13123968; doi:10.1371/journal.pone.0348093)
Supplement: Attachment — Submitted filename: Response_to_Reviewers_R2.docx [file pone.0348093.s014.docx]

**Response to Academic Editor and Reviewers**

**Manuscript ID:** PONE-D-25-61898R1

**Title:** *Ten-year natural history of visual function in Japanese patients with Leber hereditary optic neuropathy: a retrospective cohort study*

Dear Academic Editor and Reviewers,

Thank you very much for your continued time and consideration of our manuscript. We sincerely appreciate the careful reassessment of our revised manuscript. We are grateful that Reviewer #3, Reviewer #4, and Reviewer #5 indicated in the decision letter that the comments from the previous round had been addressed. We also thank Reviewer #5 for the supportive comment that our responses were appropriate and helped improve the clarity of the manuscript.

In the current round, Reviewer #3 provided additional comments as an attachment. We have carefully considered each point and made specific revisions and clarifications in the manuscript where appropriate. Below, we respond to each comment individually. We also made minor editorial corrections that were identified during this round of revision.

## Summary of editorial changes in the current round

In addition to the responses below, the following editorial corrections were made in the current round:

1. Corrected a grammatical error in the Methods section: *“we used to prespecify piecewise linear mixed-effects models”* was revised to *“we fitted prespecified piecewise linear mixed-effects models.”*

2. Corrected a minor typographical issue in the Introduction (removed an extraneous space before “phase” in “chronic- phase”).

3. Standardized the American English spelling of “enrollment” throughout the manuscript.

4. Added a brief statement in the Discussion clarifying that the sensitivity analysis restricted to m.11778G>A cases showed similar phase-specific patterns, supporting consistency of the findings across mutation types.

These revisions were editorial in nature and did not affect the study design, statistical analysis, or primary interpretation.

# Response to Reviewer #3

We thank Reviewer #3 for the additional comments provided as an attachment for the current revision round. Below, we respond to each point in the order presented.

## General

### Comment 1: Small sample size and selection bias limit generalizability.

**Response:** We agree that the modest sample size and potential selection bias are important limitations. In the revised manuscript, these points are explicitly addressed in the following locations:

• **Abstract (final sentence):** *“Given the modest sample size and selection of long-term attendees, these estimates should be interpreted as descriptive, phase-specific benchmarks rather than definitive evidence of long-term stability or ethnic differences.”*

• **Methods (final paragraph of the cohort description):** *“Thus, our analytic cohort reflects patients … who presented within 6 months of onset and remained in follow-up at our tertiary center for at least 10 years. It is therefore a long-term follow-up cohort rather than a complete inception cohort of all incident cases.”*

• **Discussion (Limitations paragraph):** We state that the study was a single-center retrospective cohort from a tertiary eye hospital, which may introduce referral and selection bias and limit generalizability, and that the sample size was modest, particularly in the late chronic phase, resulting in wide confidence intervals and limited power.

We believe the manuscript now appropriately frames the findings as descriptive benchmarks rather than population-level estimates.

### Comment 2: Wide confidence intervals reduce certainty of long-term trends.

**Response:** We agree. The wide confidence intervals for the late chronic phase are explicitly acknowledged in the following locations:

• **Abstract:** *“…with wide CIs compatible with small long-term improvement or deterioration.”*

• **Results (Table 3 and text):** The Late Chronic phase slopes are reported with their 95% CIs (+0.003 logMAR/year, 95% CI −0.007 to +0.013 overall), and the text states that *“the confidence intervals were wide and remained compatible with slight long-term improvement or deterioration.”*

• **Discussion:** *“…late-phase estimates did not show a clearly detectable directional trend and should not be interpreted as definitive stability given the wide confidence intervals and the declining number at risk over time.”*

We have used cautious language throughout to avoid overstating the certainty of long-term trends.

### Comment 3: Some sentences across sections are overly long, affecting readability.

**Response:** We have reviewed the manuscript for readability. In the current round, we identified and corrected a grammatical error in the Methods section that contributed to reduced readability (*“we used to prespecify”* was revised to *“we fitted prespecified”*). We also corrected a typographical issue in the Introduction (“chronic- phase” → “chronic phase”). We acknowledge that some sentences remain lengthy due to the technical specificity required for statistical methodology; however, we have ensured that each sentence conveys a single main idea and that the overall flow of the manuscript is as clear as possible.

## Introduction

### Comment: The Introduction could be more concise; it lacks a clear, explicit statement of study hypothesis or primary objective.

**Response:** In the revised manuscript, the study objectives are now explicitly stated in the final paragraph of the Introduction:

*“…we aimed to (i) describe the 10-year natural history of BCVA in a Japanese LHON cohort; (ii) estimate the chronic-phase slope using piecewise LMMs aligned with prior natural-history definitions; and (iii) assess consistency in a predefined subgroup defined as onset age ≥15 years and m.11778G>A subgroup.”*

Additionally, we note that this is an observational natural-history study; accordingly, we have stated research aims rather than a formal hypothesis, which we believe is appropriate for this study design. The Introduction was revised in the previous round to improve conciseness and logical flow from background to study objectives.

## Methods

### Comment 1: Rationale for phase divisions (Acute, Chronic, Late Chronic) should be briefly explained.

**Response:** The rationale for the phase definitions is provided in the Methods section, where we state:

*“Based on prior natural-history reports (e.g., REALITY and Newman et al.) [5,6], we defined the Chronic phase as 12–60 months and the Late Chronic phase as 60–120 months.”*

These references (the REALITY study and Newman et al.) provide the natural-history framework from which the phase boundaries were derived. We believe this is sufficient justification for the predefined phase divisions.

### Comment 2: Inclusion/exclusion criteria and handling of missing data are not fully detailed.

**Response:** The inclusion and exclusion criteria are specified in the Methods section. Specifically:

• **Inclusion criteria:** primary LHON mutation (m.11778G>A, m.14484T>C, or m.3460G>A), presentation within 6 months of visual loss onset, no ocular comorbidity likely to affect visual function, and continuous follow-up for ≥10 years with at least annual BCVA measurements.

• **Exclusion:** one eye was excluded due to retinal vein occlusion during follow-up (Fig 1).

• **Missing data:** The Methods section states: *“We performed available-case analyses without imputation. Linear mixed-effects models inherently accommodate unbalanced repeated measures and used all available observations under a missing-at-random assumption without imputation, and for the Kaplan–Meier analyses, participants lost to follow-up were censored at their last visit.”*

We believe the handling of missing data is now clearly described.

### Comment 3: Dense sentences reduce readability; consider splitting.

**Response:** As noted above under General Comment 3, we have corrected the grammatical error in the Methods section and improved readability where possible. We have also reviewed other dense passages for clarity.

### Comment 4: Statistical software/package not specified.

**Response:** The statistical software is specified in the Methods section:

*“Statistical analyses were performed using R statistical software version 4.4.2 (R Foundation for Statistical Computing, Vienna, Austria).”*

## Results

### Comment 1: What is Nadir BCVA? 48M and 120M?

**Response:** The definition of nadir BCVA is provided in the footnote of Table 2:

*“The nadir BCVA was the worst recorded visual acuity for each eye.”*

This is an eye-level definition: nadir BCVA represents the single worst BCVA measurement observed for each eye during the entire follow-up period. The values at 48M and 120M are not nadir values; rather, they represent the proportions of eyes achieving the prespecified BCVA thresholds (≤1.6 and ≤1.3 logMAR) at those time points. We have rechecked the manuscript and confirmed that the distinction between nadir BCVA (worst-ever) and time-specific BCVA proportions is clearly presented in Table 2 and the accompanying text.

### Comment 2: Dense numeric reporting; tables or figures could improve clarity.

**Response:** In the revised manuscript, key numeric results are summarized in tables:

• Table 1: Clinical characteristics and BCVA summary

• Table 2: Representative values of BCVA (initial, nadir, 48M, 120M proportions)

• Table 3: Piecewise LMM slopes by predefined phase

Detailed descriptive statistics are provided in S2 Table. The Kaplan–Meier cumulative achievement estimates are presented graphically in Fig 3. We believe the current combination of tables, figures, and text provides a clear presentation of the results.

### Comment 3: Percentages of eyes vs. patients sometimes unclear.

**Response:** We have rechecked the manuscript to ensure that percentages are consistently attributed to the correct unit of analysis. In particular:

• Table 1 presents patient-level characteristics (e.g., 24/27 patients [88.9%] were male).

• Table 2 and the BCVA proportion analyses are presented at the eye level (e.g., 26 eyes [49.1%]).

• The Results text now consistently specifies “eyes” or “patients” when reporting percentages.

We believe the distinction is now clearly presented throughout.

### Comment 4: Figures (LOWESS, Kaplan–Meier) need clearer referencing and brief interpretation in text.

**Response:** The revised manuscript includes in-text references to all figures:

• The LOWESS curves (Fig 2) are described as: *“The LOWESS-smoothed curves (Fig 2) showed gradual recovery after the initial decline and an approximately horizontal course thereafter, consistent with the absence of a clearly detectable late-phase trend at the group level.”*

• The Kaplan–Meier analyses (Fig 3) include detailed cumulative achievement estimates in the Results text, with explicit in-text references to each panel (A–D).

We believe the figures are now adequately referenced and briefly interpreted in the text.

### Comment 5: Functional significance of BCVA changes could be emphasized.

**Response:** The functional significance of the observed BCVA changes is addressed in the Discussion:

*“Although chronic-phase improvement was statistically detectable, the magnitude was small and may have limited functional significance; clinically, recovery—when it occurs—appears typically modest and most likely within the first few years after onset.”*

Additionally, the BCVA thresholds used in the Kaplan–Meier analyses (≤1.6 and ≤1.3 logMAR) were selected to represent clinically meaningful levels of severe visual impairment, as referenced from Newman et al. [6]. We believe the functional significance is now appropriately discussed.

### Comment 6: Consider emphasizing functional significance (e.g., BCVA ≤1.6 logMAR = threshold for legal blindness in Japan).

**Response:** We appreciate this suggestion. We note that the thresholds used in our study (≤1.6 and ≤1.3 logMAR) were selected based on prior LHON natural-history literature [6] to represent clinically meaningful levels of severe visual impairment, rather than the legal blindness criteria specific to Japan. While the Japanese legal blindness threshold is relevant, introducing a country-specific legal definition may complicate the discussion beyond the scope of this natural-history study that aims to provide internationally comparable benchmarks. We have retained the current thresholds and their clinical interpretation as defined in the Methods section.

## Discussion

### Comment 1: There could be improvement in visual acuity after certain time period across all LHON types, which was not addressed adequately.

**Response:** We agree that this is an important point. In the revised manuscript, the primary piecewise LMM analysis was performed in the overall cohort (which includes both m.11778G>A and m.14484T>C cases) and in the predefined m.11778G>A subgroup. To directly address the concern about consistency across mutation types, we performed a sensitivity analysis restricted to m.11778G>A cases (S3 Table), and the Results text states:

*“To address the potential influence of the small m.14484T>C subgroup, we repeated the primary BCVA trajectory analyses restricted to m.11778G>A cases; the phase-specific patterns were similar (S3 Table).”*

In the current round, we have added the following sentence to the Discussion to more explicitly address this point:

*“The sensitivity analysis restricted to m.11778G>A cases showed similar phase-specific patterns (S3 Table), supporting consistency of the observed trajectories across the mutation types represented in our cohort.”* [NEW]

We acknowledge that because our cohort contained no m.3460G>A cases and only 3 patients with m.14484T>C, we cannot provide mutation-specific trajectory estimates for all three primary LHON mutations. This limitation is inherent to the rarity of LHON and the composition of our cohort. We have stated this in the Limitations section.

### Comment 2: Some interpretations may overstate findings; use more cautious phrasing.

**Response:** We have reviewed the manuscript and used cautious language throughout. Key examples include:

• Abstract: *“…we did not detect a clear directional trend thereafter”* (rather than claiming stability)

• Discussion: *“…late-phase estimates did not show a clearly detectable directional trend and should not be interpreted as definitive stability”*

• Discussion: *“…overt late-phase deterioration may not be universal”* (rather than claiming no deterioration occurs)

• Conclusions: *“Rather than establishing ethnic differences, these findings offer descriptive, Japanese single-center benchmarks”*

We believe the current phrasing is appropriately cautious throughout.

### Comment 3: Limited discussion on potential biological/environmental reasons for differences from Western cohorts.

**Response:** This point is addressed in the Discussion, where we state:

*“However, we cannot fully exclude potential confounding factors, including measurement differences, environmental exposures (such as smoking), and variations in maternal haplogroup distribution.”*

Additionally, the Introduction notes that:

*“…cross-cohort differences may reflect variation in mitochondrial genetic background (e.g., haplogroup distributions), environmental exposures, and healthcare access.”*

We acknowledge that a more extensive discussion of potential biological mechanisms would be informative but would be speculative given our observational data. We have framed the observed differences as descriptive rather than mechanistic, stating that our results serve as *“single-center Japanese benchmarks that complement large international natural-history datasets.”*

### Comment 4: Clinical relevance of small chronic-phase improvements could be elaborated.

**Response:** The clinical relevance is discussed in the Discussion:

*“Although chronic-phase improvement was statistically detectable, the magnitude was small and may have limited functional significance; clinically, recovery—when it occurs—appears typically modest and most likely within the first few years after onset.”*

Furthermore, the Kaplan–Meier analyses provide complementary information about the clinical meaningfulness of BCVA changes by showing the proportions of eyes achieving clinically meaningful thresholds over time. The plateau in cumulative attainment after 60 months further supports the interpretation that most clinically meaningful recovery occurs in the earlier chronic period. We believe these discussions adequately address the clinical relevance.

# Response to Reviewer #4

We sincerely thank Reviewer #4 for the reassessment of our manuscript and for indicating that all previous comments had been addressed. We carefully rechecked the revised manuscript and the submission package in the current round. No additional substantive revisions were required in response to Reviewer #4.

# Response to Reviewer #5

We sincerely thank Reviewer #5 for the supportive reassessment and for the comment that our responses were *“perfectly appropriate and make the text much clearer.”* We carefully reviewed the manuscript again in the current round to ensure consistency and clarity throughout the revised submission. No additional substantive revisions were required in response to Reviewer #5.

# Additional checks performed in the current round

In accordance with the journal’s instructions, we also performed the following checks before resubmission:

• The reviewer comments did not include a recommendation to cite specific previously published works. We therefore made no changes to the reference list on this basis (Journal Requirement #1).

• We reviewed the reference list for completeness, accuracy, and retraction status. No retracted references were identified, and no changes were required (Journal Requirement #2).

• We verified consistency between the clean and marked-up manuscript files.

• We rechecked Supporting Information labels, captions, and in-text citations.

• We rechecked figure files against the journal’s technical requirements.

We thank the Academic Editor and all Reviewers again for their careful consideration and helpful comments. We hope that the manuscript is now suitable for publication in *PLOS ONE*.

Sincerely,

**Yasuyuki Takai, MD.**

on behalf of all authors
